# Gradually shrinking intra-abdominal desmoid tumor derived from the stomach in a young boy: a case report

**DOI:** 10.1186/s40792-017-0330-2

**Published:** 2017-04-20

**Authors:** Kazushi Miyata, Masahide Fukaya, Masato Nagino

**Affiliations:** Division of Surgical Oncology, Department of Surgery, Nagoya University Graduate School of Medicine, 65 Tsurumai-cho, Showa-ku, Nagoya, 466-8550 Japan

**Keywords:** Desmoid tumor, Stomach, Young boy

## Abstract

**Background:**

Intra-abdominal desmoid tumors, particularly those derived from the stomach, are rare. Such tumors are associated with a history of familial adenomatous polyposis (FAP), trauma, or surgical procedures in general. In addition, spontaneous shrinking of an intra-abdominal desmoid tumor is rarer. And desmoid tumors most commonly arise during the fourth decade of life.

**Case presentation:**

A 17-year-old boy with lower abdominal pain was diagnosed with a gastrointestinal stromal tumor (GIST) or a hematoma at a local hospital. He had no history of FAP, trauma, or previous surgery. Abdominal computed tomography (CT) was performed for observational purposes three times over a 9-month period. The tumor gradually decreased in size over time; however, the tumor did not shrink sufficiently to be diagnosed as a hematoma. Because there was a high possibility of a GIST from the stomach, he underwent laparotomy. Operative findings revealed that the tumor was a hard mass firmly attached to both the greater curvature of the stomach and the inferior pole of the spleen. Pathologically, the tumor was diagnosed as a desmoid tumor derived from the stomach.

**Conclusion:**

For a young boy without a history of FAP, trauma, or surgical procedures, it is difficult to define an intra-abdominal tumor near the stomach as a desmoid tumor. In such cases, surgical resection is recommended for a definitive diagnosis.

## Background

Desmoid tumors were first described in 1832 by MacFarlane [1] and account for 0.03% of neoplasms and 3% of soft tissue tumors [2]. The estimated incidence of such tumors in the general population is 2.4–4.3 per million individuals per annum [3]. Moreover, desmoid tumors are well known to be associated with familial adenomatous polyposis (FAP), with an incidence 1000-fold greater among individuals with FAP than among individuals without this condition.

Desmoid tumors are also reportedly associated with histories of trauma or prior surgery, particularly in patients with FAP [4, 5]. Desmoid tumors most commonly arise during the fourth decade of life. However, articles regarding desmoid tumors in children have been published [6–8].

We report a rare case of an intra-abdominal desmoid tumor derived from the stomach in a young patient without a history of FAP, trauma, or surgery.

## Case presentation

**Fig. 1:**
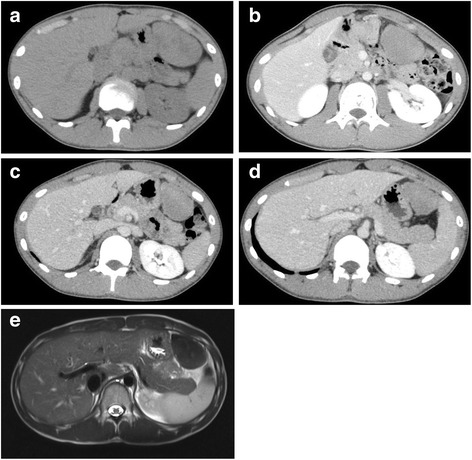
Computed tomography (CT) examinations revealed a solid mass that gradually shrank. This mass measured approximately 74 mm × 45 mm in September 2014 (**a**), 57 mm × 44 mm in December 2014 (**b**), 56 mm × 37 mm in March 2015 (**c**), and 49 mm × 34 mm in June 2015 (**d**). MRI examination revealed isointensity to the spleen on T1-weighted images and slightly inhomogeneous hypointensity on T2-weighted images (**e**)

A 17-year-old boy presented to a local hospital due to lower abdominal pain in September 2014. Abdominal computed tomography (CT) revealed a solid mass measuring approximately 74 mm × 45 mm that was attached to the greater curvature of the stomach (Fig. 1a). Esophagogastroduodenoscopy showed no remarkable findings. The tumor was diagnosed as a gastrointestinal stromal tumor (GIST) growing outward from the gastric wall.

He was referred to our hospital for management of his GIST in December 2014. He experienced no symptoms following medical examinations conducted at the local hospital. CT performed at our hospital revealed a solid mass with slightly inhomogeneous enhancement and axis lengths of 57 mm × 44 mm, which reflected a small reduction in size compared with prior CT findings (Fig. 1b). Therefore, we suspected that the mass might be a hematoma and suggested follow-up observation for the abdominal tumor.

In March 2015, 3 months after the previous examination, a third CT examination revealed that the mass had further shrunk to axis lengths of 56 mm × 37 mm and exhibited the same enhancement pattern observed previously (Fig. 1c). Because this slight shrinkage was consistent with the possibility of a hematoma, follow-up observation was continued.

Magnetic resonance imaging (MRI) performed in April 2015 demonstrated a tumor with isointensity to the spleen on T1-weighted images and slightly inhomogeneous hypointensity on T2-weighted images (Fig. 1e).

A fourth CT examination performed in June 2015 revealed that the mass had further reduced to axis lengths of 49 mm × 34 mm (Fig. 1d). Although the tumor had gradually shrunk, we could not definitively establish a diagnosis of a hematoma, as opposed to a GIST. During observation, he had no any symptoms including lower abdominal pain. Accordingly, a surgical procedure was chosen for treatment and diagnosis.

**Fig. 2:**
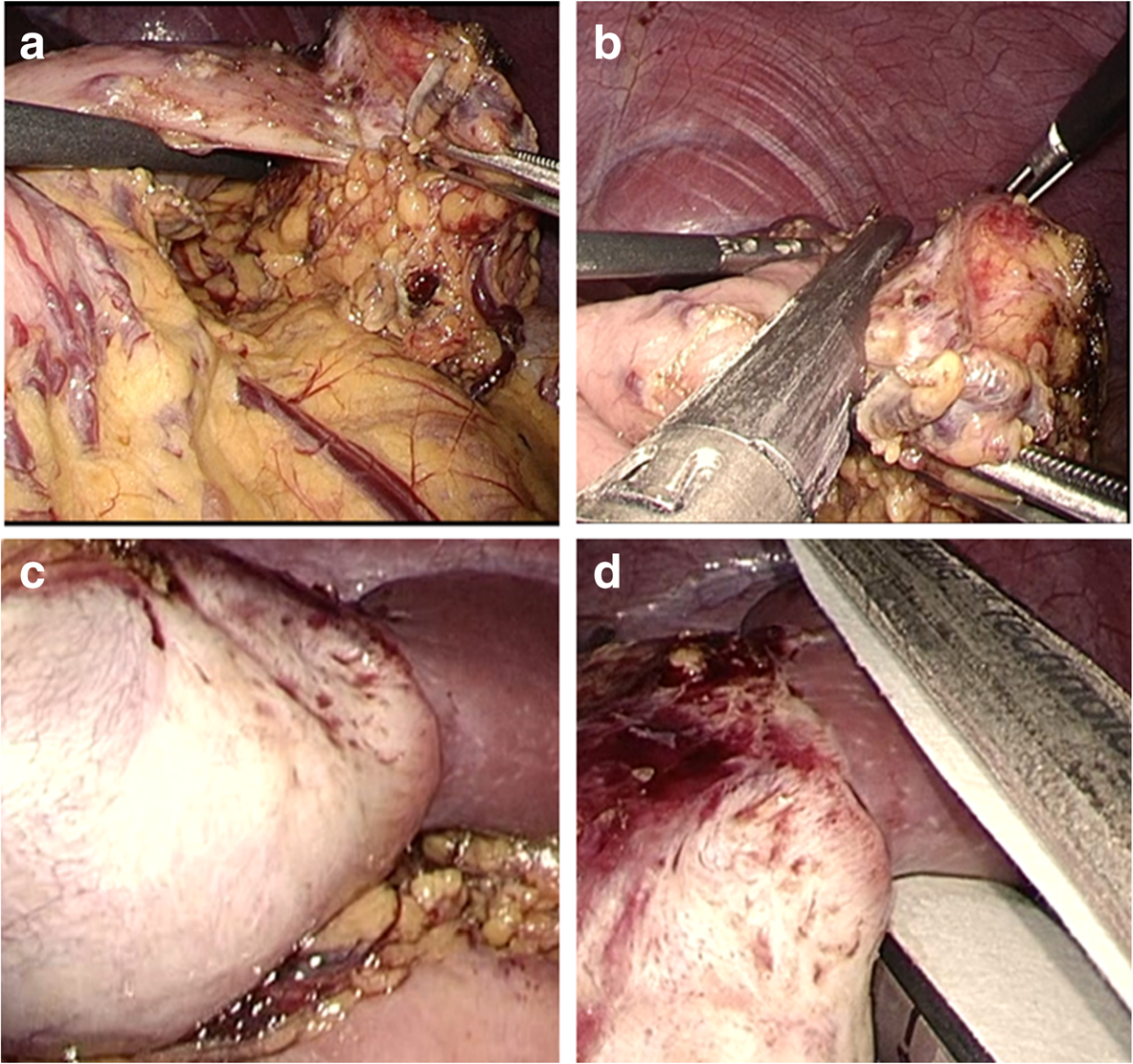
Operative findings revealed that the tumor was firmly attached to the greater curvature of the stomach (**a**) and the inferior pole of the spleen (**c**). An automatic suture device was used to detach the tumor from the stomach (**b**) and spleen (**d**)

Laparoscopic partial gastrectomy with partial splenectomy was performed by an automatic suture. Operative findings revealed that the tumor was a hard mass and was firmly attached to the greater curvature of the stomach and the inferior pole of the spleen (Fig. 2a, b). It was unclear whether this firm attachment was attributable to adhesion or direct invasion. The branches of the right and left gastroepiploic arteries fed the tumor. The feeding artery was clipped, and an automatic suture device was used to detach the tumor from the stomach and spleen (Fig. 2c, d).

**Fig. 3:**
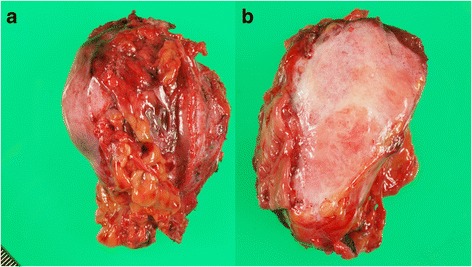
The resected specimen measured 60 mm × 50 mm × 25 mm (**a**), and the cut surface was pink and uniform (**b**)

**Fig. 4:**
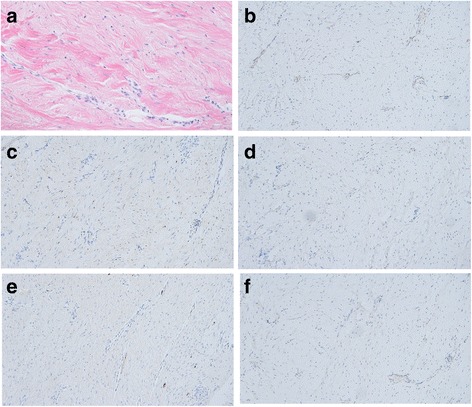
Hematoxylin and eosin staining revealed the proliferation of spindle-shaped cells and dense collagen bundles, mainly at the muscularis propria of the stomach. **a** Immunostaining indicated that tumor cells were negative for CD34 (**b**), c-kit (**c**), desmin (**d**), S-100 (**e**), and β-catenin (**f**)

Macroscopically, the tumor measured 60 mm × 50 mm × 25 mm, and the cut surface of the resected specimen was pink and uniform (Fig. 3a, b). Microscopically, the tumor exhibited the proliferation of spindle-shaped cells and dense collagen bundles, mainly at the muscularis propria of the stomach (Fig. 4a), and was diagnosed as a stomach-derived mass. Immunohistological examination showed that the tumor was negative for CD34, CD117 (C-kit), desmin, S-100, and β-catenin (Fig. 4b–f). Therefore, this tumor was eventually diagnosed as a desmoid tumor derived from the stomach.

The patient’s postoperative course was uneventful and without complications. He continues to undergo surveillance for recurrence, and no signs of recurrence have been observed for 16 months after the operation.

### Discussion

Desmoid tumors can be categorized based on three different localizations, the abdominal wall, intra-abdominal, and extra-abdominal, and the reported incidences of each type are 49, 8, and 43%, respectively. Intra-abdominal desmoid tumors are further classified into mesenteric and intrapelvic tumors. Desmoid tumors are also divided into FAP-associated and sporadic tumors. Although desmoid tumors can occur anywhere in the body, FAP-associated desmoid tumors are typically intra-abdominal. Moreover, almost all intra-abdominal desmoid tumors are associated with FAP and previous surgery. The incidence of desmoid tumors is approximately 10–15% among patients with FAP, and 12.3% of patients with desmoid tumors have been diagnosed with FAP [9].

Given the aforementioned data, the present case was extremely rare because he had an intra-abdominal, sporadic desmoid tumor without a history of FAP, trauma, or surgery. In addition, it was particularly unusual that the patient’s tumor was derived from the stomach and gradually decreased in size. To the best of our knowledge, reports of desmoid tumors derived from the stomach and articles regarding spontaneous shrinkage of an intra-abdominal desmoid tumor are rather scant [10–12]. Thus, we initially suspected that the tumor was either a GIST or a hematoma. As far as I heard the patient, there were no any abdominal trauma that caused a desmoid tumor or hematoma. However, the patient was an active high school boy, and we also hypothesized that the tumor was a hematoma that resulted from unnoticed abdominal trauma sustained when the patient was playing with his friends. However, the shrinkage of this tumor during observation puzzled us. The gradual reduction of the tumor was not consistent with a GIST. However, the size reduction would have been unexpectedly small if the tumor had been a hematoma. Therefore, the patient underwent complete resection.

In fact, despite its observed reduction in size over time, the tumor was neither a GIST nor a hematoma but rather a desmoid tumor. Diagnosis was difficult given the spontaneous decrease in tumor size. Few literature reports have described spontaneous shrinkage of desmoid tumors without treatment. A retrospective review has reported the disappearance or diminishing of five of eight tumors [12]. The reasons underlying tumor shrinkage remain unclear.

Intra-abdominal desmoid tumors have a tendency to recur locally after surgical resection, but they are not associated with the ability to metastasize [13]. The recurrence rate for desmoid tumors is high (30 to 40%) [14]. Although the recurrence rate given associated FAP that can reach 90%, the corresponding rate for sporadic desmoid tumors may only reach 10%. The optimal therapy for desmoid tumors remains controversial because large randomized studies are not abundant due to the rarity of such tumors. However, certain studies have suggested that surgical resection with negative margins is one of the most effective therapies [14–16]. In contrast, other authors have reported no relationship between surgical margins and local recurrence [17]. In any event, careful follow-up after surgery is required.

## Conclusions

It was difficult to regard the patient’s intra-abdominal tumor near the stomach as a potential desmoid tumor, in the described case involving a young boy without a history of FAP, trauma, or surgical procedures and with gradually shrinking intra-abdominal tumor. Thus, in such cases, it may be necessary to determine surgical procedures for certain diagnosis.

## Abbreviations

CT: Computed tomography
FAP: Familial adenomatous polyposis
GIST: Gastrointestinal stromal tumor
MRI: Magnetic resonance imaging
